# Identifying Risk Indicators of Cardiovascular Disease in Fasa Cohort Study (FACS): An Application of Generalized Linear Mixed-Model Tree

**DOI:** 10.34172/aim.2024.35

**Published:** 2024-05-01

**Authors:** Fariba Asadi, Reza Homayounfar, Mojtaba Farjam, Yaser Mehrali, Fatemeh Masaebi, Farid Zayeri

**Affiliations:** ^1^Department of Biostatistics, School of Allied Medical Sciences, Shahid Beheshti University of Medical Sciences, Tehran, Iran; ^2^National Nutrition and Food Technology Research Institute, Faculty of Nutrition Sciences and Food Technology, Shahid Beheshti University of Medical Sciences, Tehran, Iran; ^3^Noncommunicable diseases research center, Fasa University of Medical Sciences, Fasa, Iran; ^4^Statistical Center of Iran, Tehran, Iran; ^5^Proteomics Research Center and Department of Biostatistics, School of Allied Medical Sciences, Shahid Beheshti University of Medical Sciences, Tehran, Iran

**Keywords:** Cardiovascular diseases, Fasa Cohort Study, GLMM tree, Mixed-effect model

## Abstract

**Background:**

Today, cardiovascular disease (CVD) is the most important cause of death around the world. In this study, our main aim was to predict CVD using some of the most important indicators of this disease and present a tree-based statistical framework for detecting CVD patients according to these indicators.

**Methods:**

We used data from the baseline phase of the Fasa Cohort Study (FACS). The outcome variable was the presence of CVD. The ordinary Tree and generalized linear mixed models (GLMM) were fitted to the data and their predictive power for detecting CVD was compared with the obtained results from the GLMM tree. Statistical analysis was performed using the RStudio software.

**Results:**

Data of 9499 participants aged 35‒70 years were analyzed. The results of the multivariable mixed-effects logistic regression model revealed that participants’ age, total cholesterol, marital status, smoking status, glucose, history of cardiac disease or myocardial infarction (MI) in first- and second-degree relatives, and presence of other diseases (like hypertension, depression, chronic headaches, and thyroid disease) were significantly related to the presence of CVD (*P*<0.05). Fitting the ordinary tree, GLMM, and GLMM tree resulted in area under the curve (AUC) values of 0.58 (0.56, 0.61), 0.81 (0.77, 0.84), and 0.80 (0.76, 0.83), respectively, among the study population. In addition, the tree model had the best specificity at 81% but the lowest sensitivity at 65% compared to the other models.

**Conclusion:**

Given the superior performance of the GLMM tree compared with the standard tree and the lack of significant difference with the GLMM, using this model is suggested due to its simpler interpretation and fewer assumptions. Using updated statistical models for more accurate CVD prediction can result in more precise frameworks to aid in proactive patient detection planning.

## Introduction

 In recent decades, the rapid growth of non-communicable diseases (NCDs) has become a serious health challenge around the world.^1^ Every year, the four main NCDs [i.e. chronic respiratory diseases, cancer, diabetes, and cardiovascular diseases (CVD)] impose huge costs on the health systems of world countries.^2,3^ According to the World Health Organization (WHO), NCDs account for approximately 60% of global disability-adjusted life years (DALYs), of which about a quarter are attributable to CVD.^4^ CVD is a term referring to a range of diseases that affect the heart and blood vessels such as hypertension, ischemic heart disease, coronary heart disease (heart attack), cerebrovascular disease (stroke), heart failure, and other heart diseases.^5^ Unfortunately, the prevalence of CVD increased from 257 million in 1990 to 550 million in 2019^6^ and it is predicted that CVD will cause more than 23 million deaths globally in 2030.^7,8^ Regarding the increasing trend of incidence and mortality caused by CVD in different parts of the world, identifying factors related to this health problem can lead to early diagnosis of the patients and reduction of its burden. In this context, many studies have been conducted to determine the effective risk indicators of CVD in different populations. According to the results from these studies, hypertension, diabetes, low-density lipoprotein cholesterol (LDL-C), advanced age, high fasting plasma glucose (FPG), unhealthy diet, and being overweight or obese are among the most important risk indicators associated with CVD.^9-12^

 In recent decades, a variety of statistical approaches have been used by data analysts to determine the related indicators of different outcomes, such as CVD, diabetes, hypertension, and other health problems. Among these methods, logistic regression (LR) model, Fisher’s discriminant analysis,^13^ and area under the curve (AUC) are probably the most common approaches for identifying the factors related to different diseases and classifying the subject according to these outcomes. However, the landscape of healthcare research has transformed with the exponential growth of data and the availability of detailed medical information in recent years.^14^ Today, data warehouses are full of amazing amounts of structured and unstructured data, which have shifted the way of research from classic statistical methods to more sophisticated techniques. Machine learning (ML) has emerged as a powerful tool in this context, leveraging computational algorithms, enhanced data collection capabilities, and statistical theories in pattern recognition and prediction.^15^ ML algorithms have proven to be highly effective predictors, surpassing classic statistical models in capturing complex interactions and non-linear relationships between variables and outcomes.^16^ In ML terminology, supervised and unsupervised learning are two fundamental approaches used by data scientists in classification and clustering the study subjects. Supervised learning requires labeled data and focuses on predicting specific outcomes, while unsupervised learning aims to explore data patterns and structures without labeled examples.^17,18^ Supervised classification ML algorithms refer to a predictive modeling problem in which a class label is predicted for a given sample.^19^ The naive Bayes (NB), decision tree (DT), the k-nearest neighbor (KNN), and deep neural networks, and random forest (RF) are some of the most frequent algorithms in this field.^20^ In addition, some of these methodologies have been recently extended to more complex medical outcomes such as repeated measures (longitudinal) or clustered (panel) response data. Note that the term “prediction” is used as a keyword within statistical modeling and ML methods, irrespective of the study design. In this framework, one can construct a statistical model (based on the significant predictors) and then this model can be employed “to predict” the outcome (response variable) in the present sample as well as new cases based on their attributes.

 When the data is collected longitudinally or clustered, many ML methods may not achieve the desired level of accuracy. This failure is due to not establishing the assumption of independence among the observations which is the basic assumption required for the proper functioning of most ML algorithms.^21,22^ The integration of statistical and ML methods to develop prediction models with clustered and longitudinal data has gained significant attention in recent years. Mixed hidden Markov models (MHMMs), Hybrid RFs for high-dimensional longitudinal data, and generalized linear mixed-model (GLMM) trees are some of the advanced techniques in this field.^23^ These techniques mostly result in better prediction performance due to matching the data structure. Among the mentioned methods, GLMM tree has gained prominence due to its versatility across various applications and interpretability.^21,24,25^

 In recent years, many studies have been conducted in connection with the identification of CVD risk indicators using ML methods. In 2020, Yang et al examined a CVD prediction model based on RF, CART, NB, Bagged Trees, and Ada Boost in eastern China.^26^ In 2023, Subramani et al investigated the integration of deep learning with ML methods including SVM, KNN, LR, XGBoost, NB, LR, and DT.^16^ In another study in 2022, the researchers employed the multi-layer perceptron and KNN techniques for detecting CVD patients using data publicly available in the University of California Irvine repository.

 As mentioned previously, numerous studies have been conducted for CVD prediction and identifying the factors affecting the occurrence of CVD in different parts of the world. However, considering that Iran has the highest burden of CVD in the Eastern Mediterranean region, and so far, there has been limited information on determining the indicators of this disease in high-volume data collected in clusters, we decided to conduct the current study on the data from the Fasa cohort with the following goals: First, to determine the prevalence of CVD in the study population and second, to use tree-based methods in this population for predicting CVD patients based on some of its most important indicators. The authors hope that the findings of their research will be useful in identifying people at risk of developing CVD and lowering the burden of this disease in the population.

## Materials and Methods

###  Fasa Cohort Study

 In this study, data from the baseline phase of the Fasa Cohort Study (FACS) was utilized. The FACS has been designed to examine and evaluate the health conditions and risk factors that contribute to the increased vulnerability of rural inhabitants to NCDs in the Fasa region. With a population of about 250 000, Fasa is a county located in the eastern part of the Fars province, southern Iran. This cohort included 10,146 participants aged 35‒70 years from Sheshdeh and Qarabalag region, the suburb of Fasa city and its 29 satellite villages. The villages (rural regions) were considered clusters (incorporated into the model as random effects). Demographic characteristics, medical information, and history of nutrition and lifestyle data were collected for each participant using standard questionnaires. The inclusion criteria of this study were Iranian nationality, at least one year of residence in the region, age between 35 and 70 years, willingness to participate, and the ability to communicate verbally. Also, the exclusion criteria from the study were non-attendance after three phone calls. Before data gathering, written consent was obtained from all participants. More detailed information about the design of the FACS and its participants can be found elsewhere.^27^

###  Main Outcome and Potential Predictors

 The outcome variable was defined as the presence of CVD in the baseline phase of the FACS. In this study, the participants with heart failure or ischemic heart disease were considered as those with CVD. In addition, the demographic characteristics of the participants (such as age, gender, marital status, and level of education), time to wake up in the morning, time to sleep at night, wealth score index (WSI), metabolic equivalent of task (MET), body mass index (BMI), the dietary inflammatory index (DII), biochemical markers (LDL, ALP, GGT, total cholesterol, glucose, triglyceride), waist-to-height ratio, having other diseases (like diabetes, hypertension, thyroid problems, chronic headaches, depression, fatty liver), smoking status, alcohol consumption, tobacco use, history of diseases including diabetes, hypertension, myocardial infarction (MI), and cardiac disease in the first- and second-degree relatives were considered as the potential indicators for CVD in the data analysis process.

###  Statistical Analysis

 After excluding individuals with at least 50% missing data in the input variables, the analysis focused on the data from 9499 subjects where less than 1% of the data for each variable were missing and subsequently imputed. For descriptive purposes, the central tendency and dispersion indices were calculated for the quantitative variables, and the frequency distribution was reported for the qualitative factors. The relationship between qualitative variables was assessed using the chi-square test, and the independent samples t-test was used to compare the means of quantitative variables between two independent groups. In the second step, an initial screening of the predictors was performed using a multivariable mixed-effects LR model using the glmer package. In this stage, Z-normalization was used for numerical variables. Variables with *P* values less than 0.1 were considered as potential related indicators of CVD. Regarding this, 13 variables (out of 32 preliminary indicators) remained in the final modeling process. WSI and MET were considered as the confounding variables. In the third step, the ordinary tree with CART (classification and regression tree) algorithm was fitted to the data using the rpart package.

 Then, the GLMM tree was fitted to the data using the glmertree package. We used the post-pruning method for the overfitting problem in the decision tree. This method is also done within the GLMM tree algorithm. We used the 10-fold cross-validation and the train-test split technique to compare the performance of the fitted models. It is worth noting that the precision of ML models is impacted by the choice of the cutoff point used for classifying observations; this is especially crucial when there are varying cluster sizes in both the training and testing datasets. In this study, the cutpoint was selected as the value at which, in the training set, the proportion of observations assigned to class 1 was closest to the true proportion of class 1. Finally, the obtained results of GLMM tree were compared with the findings from the ordinary tree and the generalized mixed-effects logistic regression model (GLMM). All statistical analyses were performed using the RStudio software (version 2023.06.0). *P* values less than 0.05 were considered statistically significant.

###  Generalized Linear Mixed-Effects Tree Model

 The GLMM is one of the statistical approaches frequently used in modeling longitudinal and clustered data. This family of statistical models enables data analysts to take into account the correlation between the outcome data by adding random terms to the linear or non-linear models.

 Generally, a GLMM can be defined as:


$$g\left(\mu_{i}\right)=\eta_{i}=X_{i}\beta +Z_{i}b_{i},\;b_{i}∼N\left(0,D\right),\;i=1,2,...,n$$

where *g*(*μ*_i_) is a known link function, *X*_i_*β* is the fixed-effects component, *Z*_i_*β*_i_ is the random-effects component, *D* is the variance-covariance matrix of the random term, and *I* represents the cluster number.^28^

 Although GLMMs are powerful statistical tools for identifying the predictors associated with different types of medical outcomes, they do not provide direct guidance for clinical decision-making. Compared to traditional generalized linear (Mixed) models (GL(M)Ms), tree-based methods provide a more explicit framework for decision-making processes.^29^ The basic idea behind the GLMM tree (which is an extension of the decision tree approach) is to substitute the linear structure employed for modeling the fixed-effects component (*X*_i_*β*) in the GLMM’s linear predictor with a standard tree structure, while retaining a linear structure for the random component, consistent with the GLMMs. In this context, the GLMM tree can be written as:


$$\begin{array}{l} g\left(\mu_{i}\right)=\eta_{i}=f\left(X_{i}\right)+Z_{i}b_{i},\;b_{i}∼N\left(0,D\right),\;i=1,2,...,n\\ ,\;N=\sum_{i=1}^{n}n_{i}\;. \end{array}$$

Where *n*_i_ is the number of observations in cluster *I*, *N* represents the total number of observations, and other quantities are the same as the above-mentioned equation.^28^ This approach correctly accommodates the clustered or longitudinal structure and potential correlation between observations. Furthermore, the GLMM tree method enhances flexibility and obviates the need for assumptions related to linear associations or normally distributed residuals.^30^ The GMERT model enables the prediction of responses for two types of new observations: (1) those belonging to a cluster used in model fitting and (2) those from a cluster not part of the model’s training data. When predicting the response for a new observation in category 1, both its fixed component prediction and the predicted random part specific to its cluster are considered, resulting in a cluster-specific estimate. For new observations in category 2, only the fixed component prediction is used, with the random part set to 0.^28^

## Results

 In this study, we analyzed data from a total sample of 4199 men (44.20%) and 5300 women (55.80%) with a mean (SD) age of 48.95 (9.47) years. In terms of educational status, about half of the participants (47.09%) were illiterate, 5259 subjects (50.87%) had a high school diploma or less, and the rest had academic education. The mean (SD) body mass index of the participants was 25.69 (4.83) and about 89% of them were married. In the first step of data analysis, we described the general characteristics of the sample by the presence of CVD. According to Table 1, the prevalence of CVD at the baseline phase of the study was 11.10% (9.12% in males and 12.70% in females). About 28.95% of the participants with hypertension had CVD. The mean (SD) age of those with and without CVD was 55.44 (9.05) and 48.14 (9.20), respectively. The univariate statistical analyses show that there was a significant relationship between the participants’ age, gender, educational level, BMI, marital status, total cholesterol, tobacco use, glucose, smoking status, history of MI in first-degree relatives, history of cardiac disease in first and second-degree relatives, and having other diseases (hypertension, depression, chronic headaches, and thyroid problems) with the presence of CVD (Table 1).

**Table 1:** General Characteristics of the Sample by Presence of CVD

**Variable**	**Category**	**Without CVD**	**With CVD**	***P *** **Value**	**Total**
		**No. (%)**	**No. (%)**		**No. (%)**
Gender	Male	3816 (90.88)	383 (9.12)	< 0.001*	4199 (44.20)
	Female	4627 (87.30)	673 (12.70)		5300 (55.80)
Marital Status	Single	320 (97.86)	7 (2.14)	< 0.001*	327 (3.44)
	Married	7543 (89.23)	910 (10.77)		8453 (88.98)
	Widowed	490 (78.90)	131 (21.10)		621 (6.54)
	Divorced	90 (91.84)	8 (8.16)		98 (1.04)
Education	Illiterate	3771 (84.31)	702 (15.69)	< 0.001*	4473 (47.09)
	Elementary school	2849 (91.99)	248 (8.01)		3097 (32.60)
	Middle school	1151 (94.03)	73 (5.07)		1224 (12.89)
	High school Diploma	487 (95.30)	24 (4.70)		511 (5.38)
	Above diploma	185 (95.36)	9 (4.64)		194 (2.04)
Age	Mean ± SD	48.14 ± 9.20	55.44 ± 9.05	< 0.001**	48.95 ± 9.47
BMI	Mean ± SD	25.59 ± 4.83	26.49 ± 4.75	< 0.001**	25.69 ± 4.83
Total cholesterol	Mean ± SD	186.41 ± 38.38	181.01 ± 43.71	< 0.001**	185.81 ± 39.04
Glucose	Mean ± SD	91.86 ± 28.06	101.72 ± 38.62	< 0.001**	92.95 ± 29.58
Hypertension	No	7054 (93.50)	490 (6.50)	< 0.001*	7544 (79.42)
	Yes	1389 (71.05)	566 (28.95)		1955 (20.58)
Thyroid disease	No	7739 (89.42)	916 (10.58)	< 0.001*	8655 (91.11)
	Yes	703 (83.39)	140 (16.61)		844 (8.89)
Chronic headaches	No	7158 (89.67)	825 (10.33)	< 0.001*	7983 (84.04)
	Yes	1285 (84.76)	231 (15.24)		1516 (15.96)
Depression	No	7902 (89.26)	951 (10.74)	< 0.001*	8853 (93.20)
	Yes	541 (83.75)	105 (16.25)		646 (6.80)
Smoking status	No	6212 (89.35)	740 (10.65)	0.017*	6952 (73.19)
	Yes	2231 (87.59)	316 (12.41)		2547 (26.81)
Tobacco use	No	7943 (88.62)	1020 (11.38)	< 0.001*	8963 (94.38)
	Yes	498 (9326)	36 (6.74)		534 (5.62)
MI history in 1^st^ degree family	No	6798 (90.29)	731 (9.71)	< 0.001*	7529 (79.29)
	Yes	1642 (83.48)	325 (16.52)		1967 (20.71)
CVD history in 1^st^ degree family	No	5005 (91.12)	488 (8.88)	< 0.001*	5493 (57.83)
	Yes	3438 (85.82)	568 (14.18)		4006 (42.17)
CVD history in 2^st^ degree family	No	7147 (89.24)	862 (10.76)	< 0.001*	8009 (84.31)
	Yes	1296 (86.98)	194 (13.02)		1490 (15.69)

WSI,wealth score index; MET, metabolic equivalent of task; CVD, cardiovascular disease; MI, myocardial infarction. * The chi-square test; **The independent samples t-test.

 In the second step, a multivariable mixed-effects LR model was employed to determine the risk indicators of CVD in the population under study. Table 2 shows the obtained estimates. According to these results, variables age, total cholesterol, marital status, smoking status, glucose, tobacco use, history of cardiac disease in first and second-degree relatives, history of MI in first-degree relatives, and having other disease (including hypertension, depression, chronic headaches, and thyroid problems) were significantly related to the presence of CVD in this cohort (*P* < 0.05).

**Table 2:** Mixed-Effects Logistic Regression Analysis for Assessing the Concurrent Relationship between Predictors and CVD

**Variable**	**Subgroup**	**Estimate**	**Odds Ratio**	***P *** **Value**
Marital Status	Married	1.04	2.83	0.010
	Widowed	1.06	2.90	0.011
	Divorced	0.85	2.34	0.138
	Single	Reference category		
Hypertension	Yes	1.19	3.32	< 0.001
	No	Reference category		
Chronic headaches	Yes	0.36	1.44	< 0.001
	No	Reference category		
Depression	Yes	0.48	1.63	< 0.001
	No	Reference category		
Thyroid disease	Yes	0.26	1.30	0.038
	No	Reference category		
Smoking status	No	-0.38	0.68	< 0.001
	Yes	Reference category		
Tobacco use	No	-0.63	0.53	0.008
	Yes	Reference category		
MI history in 1^st^ degree family	Yes	0.32	1.38	0.003
	No	Reference category		
CVD history in 1^st^ degree family	Yes	0.52	1.68	< 0.001
	No	Reference category		
CVD history in 2^nd^ degree family	Yes	0.61	1.84	< 0.001
	No	Reference category		
Age	---	0.69	1.99	< 0.001
Total cholesterol	---	-0.24	0.79	< 0.001
Glucose	---	0.08	2.35	0.007
WSI		-0.03	0.97	0.521
MET		-0.15	0.85	0.001

WSI,wealth score index; MET, metabolic equivalent of task; CVD, cardiovascular disease; MI, myocardial infarction.

**Figure 1:**
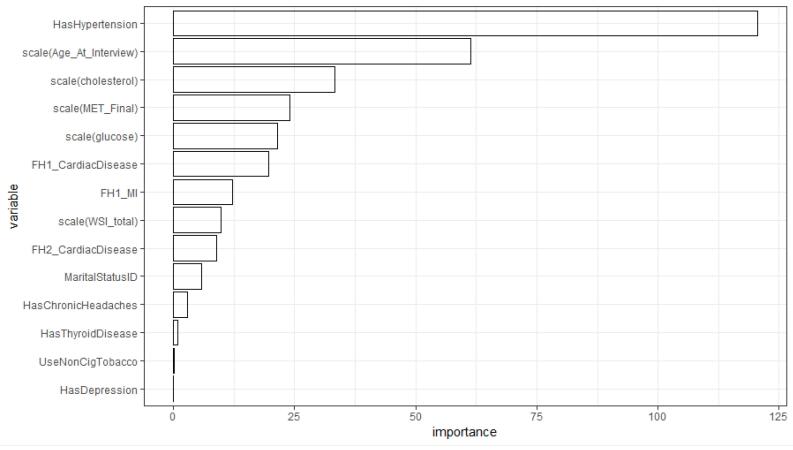
Importance Index of Indicators in the Tree Model for CVD Prediction

**Figure 2:**
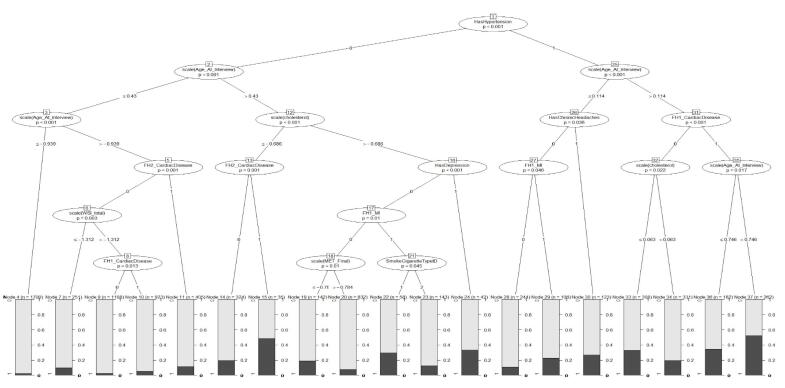
GLMM Tree for Predicting CVD (black and gray represent individuals with and without CVD, respectively )

 We also fitted an ordinary decision tree and a GLMM tree to the data. Figure 1 shows the variable importance indices obtained from fitting the decision tree model. According to these results, hypertension was the most important risk indicator for the presence of CVD (Figure 1). In addition, the variables age, total cholesterol, MET, glucose, and CVD history in first-degree family members showed higher importance compared to the other variables. Also, according to the GLMM tree in Figure 2, hypertension was identified as the most important risk indicator of CVD in the root node. Patients can be assigned to one of the terminal nodes based on their risk factors. For example, in node 70, patients have the following criteria: “Hypertension = 1”, “Age > 50”, “FH1_CardiacDisease = 0”, and “cholesterol > 188.2”. In this category, the observed proportion of cases with CVD is approximately 0.20.

**Figure 3:**
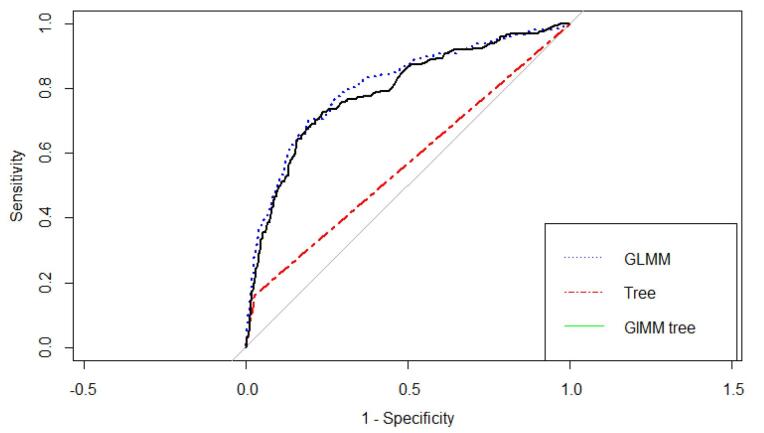
Receiver Operating Characteristic Curve for Different Machine Learning Models

 Finally, we estimated the predictive power indices of the ordinary decision tree, GLMM, and GLMM tree. According to the results in Table 3, it seems that the three models exhibit reasonably good accuracy in CVD prediction. While the tree model shows the best specificity (81%), it presents the lowest sensitivity (65%) compared to the other two models. Figure 3 also shows that the AUC of the GLMM [AUC = 0.81 (0.77,0.84)] is similar to the GLMM tree [AUC = 0.80 (0.76,0.83)], and the two models exhibited superiority over the tree model [AUC = 0.58(0.56,0.61)].

**Table 3:** Predictive Power Indices for Comparing the Results From Ordinary Tree, GLMM, and GLMM Tree

**Model**	**Sensitivity**	**Specificity**	**Accuracy**	**AUC**	**Log-likelihood**	***P *** **Value**
Tree	0.65	0.81	0.80 (0.78,0.81)	0.58 (0.56,0.61)	-	-
GLMM	0.72	0.76	0.75 (0.72,0.76)	0.81 (0.77,0.84)	-2204.4	*
GLMM Tree	0.73	0.74	0.74 (0.72,0.76)	0.80 (0.76,0.83)	-2206.2	0.999

AUC, under the curve. *Reference.

## Discussion

 Cardiovascular disease is a general term for a group of disorders that affect the heart and blood vessels. Extensive research within the medical field has focused on this topic due to its significant impact on global health and the need for better understanding of the complex interplay between risk factors and the pathogenesis of CVD. In this current research, the goal was to diagnose CVD and its risk factors using the GLMM tree. Decision tree methods were chosen for their non-parametric nature, which means they do not rely on assumptions like linear associations or normally distributed residuals. Additionally, decision-tree methods allow for the inclusion of a wide range of potential predictor variables, even when the number of variables exceeds the number of observations.^29^ Based on the findings of mixed effect LR and GLMM tree analysis, it is evident that high blood pressure exerts the most substantial influence on the occurrence of heart disease. This finding has been confirmed in many studies.^31-34^ In a cohort study of 4.3 million adults aged 30 to 90 years in the United Kingdom, the findings showed that the risk of CVD escalated consistently with increasing levels of baseline systolic blood pressure (SBP) and diastolic blood pressure (DBP) above average levels of 115 and 75 mmHg, respectively. Specifically, for every 20 mmHg rise in SBP and 10 mm Hg rise in DBP, the risk of developing CVD doubled.^35,36^ The increasing prevalence of hypertension may be attributed to socioeconomic and lifestyle changes, urbanization, and increased life expectancy. However, lack of awareness and inadequate control of high blood pressure in Iran and other low- and middle-income countries exacerbate the problem.^7^ This underscores the critical importance of managing and controlling blood pressure levels to mitigate the risk of developing cardiovascular health conditions.

 According to the findings, age and family history of disease including hypertension, MI, and CVD were also found to be very important factors in heart disease, which is consistent with previous studies.^37-39^ For example, Ambroziak et al observed statistically significant differences across the MI < 50, MI ≥ 50 and no-MI < 50 groups in the prevalence of CVD events at every age in family members (the first- and the second-degree relatives).^37^ Although age is an uncontrollable factor, emphasizing the management of modifiable risk factors in adults can effectively decrease the risk of developing the disease. Furthermore, identifying a positive family history of the listed diseases, provides healthcare providers with an opportunity to highlight the increased risk of developing CVD at a young age. This insight serves as a compelling incentive for patients to prioritize improving their adherence to healthy lifestyles and medical regimens.

 In our research, the analysis revealed a significant association between marital status and the occurrence of heart disease. After adjusting for other risk factors, the odds ratio of CVD in married (OR = 2.83) and widowed (OR = 2.90) individuals was approximately three times that of single people. This finding aligns with a 2019 cohort study that included 9737 Iranian adults in the range of 30–89 years with 12 years of follow-up. The study focused on the relationship between marital status and major clinical outcomes. Their results indicated that individuals who had never been married exhibited a significantly lower risk of CVD with a hazard ratio of 0.20 (95% confidence interval: (0.09‒0.44).^40^ However, this finding contrasts with a 2018 meta-analysis conducted by Wang et al,^41^ which stated that unmarried people are at an increased risk of CVD compared to married individuals. One possible reason for this inconsistency could be the difference in classifying marital status. In Wang’s study, the unmarried category encompassed those who were never married, divorced, or widowed. In contrast, in our study, the “single” category only included individuals who had never been married. This divergence in classification may have contributed to the disparity in the results observed between the studies. Our results revealed a significant correlation between specific conditions such as depression, chronic headaches, and thyroid issues and increased risk of CVD. These findings are in line with previous research.^42-45^ For instance, Silverman et al stated that there is a causal link between depression and adverse cardiac events, including sudden cardiac death.^42^ Additionally, a study in China suggests a potential interaction between reduced sensitivity to thyroid hormones and UA metabolism, leading to an elevated risk of CVD.^43^ Furthermore, in 2018, a meta-analysis extracted from 16 cohorts including 1 152 704 individuals showed that individuals with migraines face a 1.4-fold higher risk of cardiovascular and cerebrovascular events, MI, and stroke.^46^ This suggests that all types of headaches may be associated with metabolic risk factors and serve as indicators of cardiovascular risk. It is important to acknowledge these connections and pursue further research to deepen our understanding of these associations and their underlying mechanisms. By recognizing the connection between these conditions, clinicians can take a more comprehensive approach to managing patients, addressing both their mental health and cardiovascular well-being.

 In this study, the prevalence of CVD was found to be 11.1% which was aligned with the results of the Global Burden of Disease Study 2015, Based on this study, Iran had one of the highest rates of CVD in the world, with more than 9000 cases of CVD per 100 000 individuals.^7^ In contrast, Hinton et al conducted a study in 2018 within an English primary care sentinel network, where they reported a prevalence of 21.3% for CVD in a population of 1 275 174 individuals.^47^ It is important to note that the Hinton and colleagues’ study included participants aged 18 and above, whereas the current study specifically examined individuals aged 35 and above.^47^ Furthermore, differences in lifestyle, weather, and climate between the study populations may have also contributed to the variation observed in CVD prevalence.

 Based on the findings of this study, the predictive power indices of the GLMM were obtained similarly to the GLMM tree. These two models exhibited acceptable and nearly equal sensitivity and specificity ( ≥ 70%) and the AUC index was also around 80%, indicating a very high probability of correct classification. In addition, both models outperformed the tree model, which shows that the use of the mixed model increases the efficiency of the model due to considering the correlation among observations in clustered and longitudinal studies. In the study by Salvatore et al, which was conducted in 2021 with the aim of investigating the determinants influencing the costs of CVD in the health service in Italy’s Apulia region, the findings also revealed that GLMM showed superior performance compared to GLM, emphasizing the importance of incorporating random effects to enhance the model’s accuracy.^48^ Furthermore, there was no significant difference between the GLMM tree and GLMM, which is consistent with Fokkema and colleagues’ study. In their study in 2021, Fokkema et al compared GLMM trees with RFs and traditional GLMM. The results of their study showed that Traditional GLMMs exhibited slightly higher predictive accuracy compared to GLMM trees, while RFs showed relatively lower predictive accuracy in comparison to both traditional GLMMs and GLMM trees.^29^ The primary benefit of decision tree techniques is that they make few assumptions about data distribution. Although the AUC of the GLMM tree was slightly lower than the GLMM, it should be noted that GLMMs rely on assumptions like a linear relationship between predictors and outcome variable and as well as normal distribution for model residuals. Deviations from these assumptions can result in misleading relationships, particularly in mixed-effects models, whereas we do not need these assumptions in the GLMM trees methodology. As mentioned, the GLMM tree is preferable to the GLMM due to its graphical form and easier interpretation for the general public and the lack of presuppositions. In addition, compared to other ML algorithms used for prediction, single decision trees possess a distinct advantage in terms of interpretability.^29^ The GLMM tree clearly shows how likely the disease is according to the patient’s characteristics. For example in this data, individuals who are over 50 years of age and have hypertension, cholesterol levels of 189.9 or higher, as well as a family history of CVD and MI in their first-degree relatives, are more likely to have CVD disease ( ≥ 60%). In other words, in this group, approximately 60% of observed people have CVD.

 In summary, according to the GLMM tree, age, marital Status, total cholesterol, glucose, having other diseases (hypertension, chronic headaches, depression, thyroid disease), family history of disease including MI in first-degree relatives, and CVD in first- and second-degree relatives were important variables in CVD disease, among which hypertension was identified as the foremost risk factor associated with CVD disease. Since CVD is the leading cause of mortality and DALYs in Iran, effectively identifying the risk factors associated with CVD using suitable models can yield more precise results and facilitate proactive planning for early detection and cost-effective management of the disease. Employing such models can significantly contribute to reducing both the financial burden and mortality rates attributed to CVD.
